# Benign thyroid disease and the risk of breast cancer: An updated systematic review and meta-analysis

**DOI:** 10.3389/fendo.2022.984593

**Published:** 2022-10-12

**Authors:** Mingyue Han, Yao Wang, Yuanhui Jin, Xue Zhao, Haiying Cui, Guixia Wang, Xiaokun Gang

**Affiliations:** ^1^ Department of Endocrinology and Metabolism, The First Hospital of Jilin University, Changchun, China; ^2^ Department of Orthopedics, The Second Hospital Jilin University, Changchun, China; ^3^ Hospital Office, Meihekou City Central Hospital, Meihekou, China

**Keywords:** benign thyroid disease, breast cancer, aggressiveness, meta-analysis, female

## Abstract

**Background:**

The correlation between benign thyroid disease (BTD) and breast cancer (BC) has long been discussed. However, the definite relationship and potential mechanism between them are still disputed. The current meta-analysis aimed at performing a comprehensive assessment of the relationship between different types of benign thyroid disease and the risk of breast cancer, furthermore, assessing whether benign thyroid disease exerts an influence on the aggressiveness of breast cancer.

**Method:**

A systematic literature search (PubMed, Web of Science, MEDLINE, and Embase databases) identified studies to evaluate the correlation between BTD and BC risk. Data were analyzed using version 16.0 STATA software, including the odds ratio (OR) and its corresponding 95% confidence intervals (CIs). Publication bias and quality assessment were conducted for the included studies.

**Result:**

Overall, 18 studies involving 422,384 patients with BTD were incorporated. The outcome showed that autoimmune thyroiditis (OR: 2.56, 95%CI: 1.95–3.37, I^2 =^ 0.0%, p=0.460), goiter (OR: 2.13, 95%CI: 1.19-3.79, I^2 =^ 80.6%, p=0.000), and Graves’ disease (OR: 5.01, 95%CI: 1.49-16.82, I^2 =^ 0.0%, p=0.358) was connected with a higher risk of BC. Both hypothyroidism (OR: 0.82, 95%CI: 0.64-1.04, I^2 =^ 85.0%, p=0.000) and hyperthyroidism (OR: 1.07, 95%CI: 0.93-1.24, I^2 =^ 24.9%, p=0.206) had no significant association with the risk of BC. Additionally, the pooled analysis showed no apparent correlation between BTD and aggressiveness of BC. However, subgroup analysis indicated a positive relationship between BTD and aggressiveness of BC in the Europe subgroup (HR: 2.05, 95%CI: 1.32-3.17, I^2 =^ 86.4%, p=0.000).

**Conclusion:**

Autoimmune thyroiditis, goiter, and Graves’ disease are connected with an increased risk of BC. Furthermore, subgroup analysis suggested that BTD increases the aggressiveness of BC in the European population geographically. Nevertheless, further research is needed to prove these discoveries.

## Introduction

In 2020, the latest data from the International Agency for Research on Cancer (IARC) of the World Health Organization indicates that the number of breast cancer (BC) patients has increased to 2.26 million, becoming the most common cancer around the world (1, 2). Thus, identifying the possible risk factors and making timely prevention to reduce the incidence of BC is of great significance. Many risk factors, for instance, aging, gender, estrogen, family history, gene mutation, and unhealthy living habits, have been proven to increase the probability of BC (3). Since both thyroid and breast are regulated by the hypothalamus-pituitary-gland axis, there are likely some internal relationships and mutual influences between BC and benign thyroid disease (BTD).

It was Schottenfeld et al (4) who first proposed the connection between BTD and BC. Although it failed to prove the relationship between them, it provided new insights for later researchers. However, the existing studies showed inconsistent results. Several studies have shown that BTD increased the risk of BC (5–15), while some studies have shown that BTD decreased the risk of BC (16, 17). Besides, some studies found no connection between BTD and the risk of BC (15, 18–22). Consequently, whether BTD would increase the risk of BC needs further investigation.

The previous meta-analysis by Hardefeldt et al. confirmed autoimmune thyroiditis (AITD), goiter, and anti-thyroid antibody were positively associated with the risk of BC was published in 2012 (23). However, the influence of Graves’ disease (GD), hypothyroidism, and hyperthyroidism on the risk of BC hasn’t been illuminated yet and the relationship between BTD and the aggressiveness of BC is unknown. Furthermore, the recently published high-quality clinical research (5, 6, 13, 17) is not incorporated in the previous meta-analysis (23), which adds justification to the performance of the current study.

Therefore, our updated meta-analysis aims to systematically review and evaluate the impact of different types of BTD on the risk of BC which contains the most recent works and subgroup analysis was also conducted based on different regions. Compared to existing research, we further identify the underlying relationship between BTD and aggressiveness of BC based on different aggressiveness markers, and it is of great significance in clinical practice.

## Methods

### Search strategy

A systematic and comprehensive search for relevant literature was performed using PubMed, Web of Science, MEDLINE, and Embase databases up to date to August 2020. The following keywords were used to select relevant studies from databases: “benign thyroid disease” or “autoimmune thyroiditis” or “goiter” or “hyperthyroidism” or “hypothyroidism” or “graves” AND “breast disease” or “breast neoplasms” or “mammary cancer” AND “risk” or “incidence”. In addition, we identified potential series manually by searching the references lists and citations of retrieved papers and relevant systematic reviews, we also retrieved additional studies from PubMed option ‘Related Articles’ at the same time.

### Study selection

The titles, abstracts, and full-text studies were independently reviewed by two authors (Mingyue Han and Tong Zhou) to determine qualification for inclusion. Inclusion criteria are: (1) included patients should be older than 18 years old when first diagnosed with BTD; (2) the endpoint of included patients was the diagnosis of BC and no antitumor therapy was given; (3) the diagnosis criteria of BTD were shown in Additional file 1 (**Supplement Figure 1** **)**; (4) BC was diagnosed based on histopathological criteria (24); (5) an internal control group can be used for OR calculation; (6) we only included studies published in English in our paper, in case of missing information, we also reviewed the abstracts of non-English papers. No restrictions have existed on language or study size.

However, the studies were excluded based on the following conditions: (1) patients with a family history of BC; (2) BTD patients with a medication history that affect hormone levels; (3) the populations and the databases of the articles were duplicated from other published articles; (4) unable to obtain full-text or insufficient information was provided for quality assessment of the literature; (5) reviews, case reports, conference abstracts, letters, or meta-analyses.

### Data extraction and quality assessment

Data from studies were separately extracted by two reviewers (Yuanhui Jin and Xue Zhao) based on the inclusion and exclusion criteria as mentioned above, discussion and consensus were achieved by a third inspector (Haiying Cui) when disagreement happened. We acquired the following items from each study included in the current meta-analysis: first author, year of publication, region, study design, numbers of BTD and BC, mean age, and type of BTD. The quality of the cohort studies was assessed by the Newcastle-Ottawa Scale (NOS) (25) which included three categories: (1) selection of research object ranged from 0 to 4 points; (2) the baseline comparability ranged from 0 to 2 points; (3) clinical outcome ranged from 0 to 3 points. The NOS score is more than 6 was considered to be high-quality literature. A score of 6 is considered to be of medium quality, while a score of less than 6 is considered to be of low quality. The quality evaluation of the articles included in this meta-analysis was shown in **Supplement Figure 2**.

### Statistical analysis

A pooled odds ratio (OR) and its accompanying 95% confidence intervals (CIs) were calculated for the impact of different kinds of BTD on the probability of developing BC through a random-effects model. To further investigate the influence between BTD and the aggressiveness of BC, a pooled hazard ratio (HR) and their accompanying 95% CIs were calculated. Cochran’s Q statistic with a *p-*value < 0.1 was used to evaluate heterogeneity, which indicated significant heterogeneity. When *I²* > 50% (*p* < 0.05), a random-effect model was used for the study and was considered heterogeneous. Conversely, when *I²* < 50%, it showed low or medium heterogeneity and was evaluated using a fixed-effect model (26, 27). Data were displayed in the form of forest plots. When the *P*-value < 0.05, it was considered statistically significant. All analyses and calculations were performed in Stata version 16.0 (StataCorp, College Station, Texas, USA).

## Result

### Search results and study characteristics

**Figure 1:**
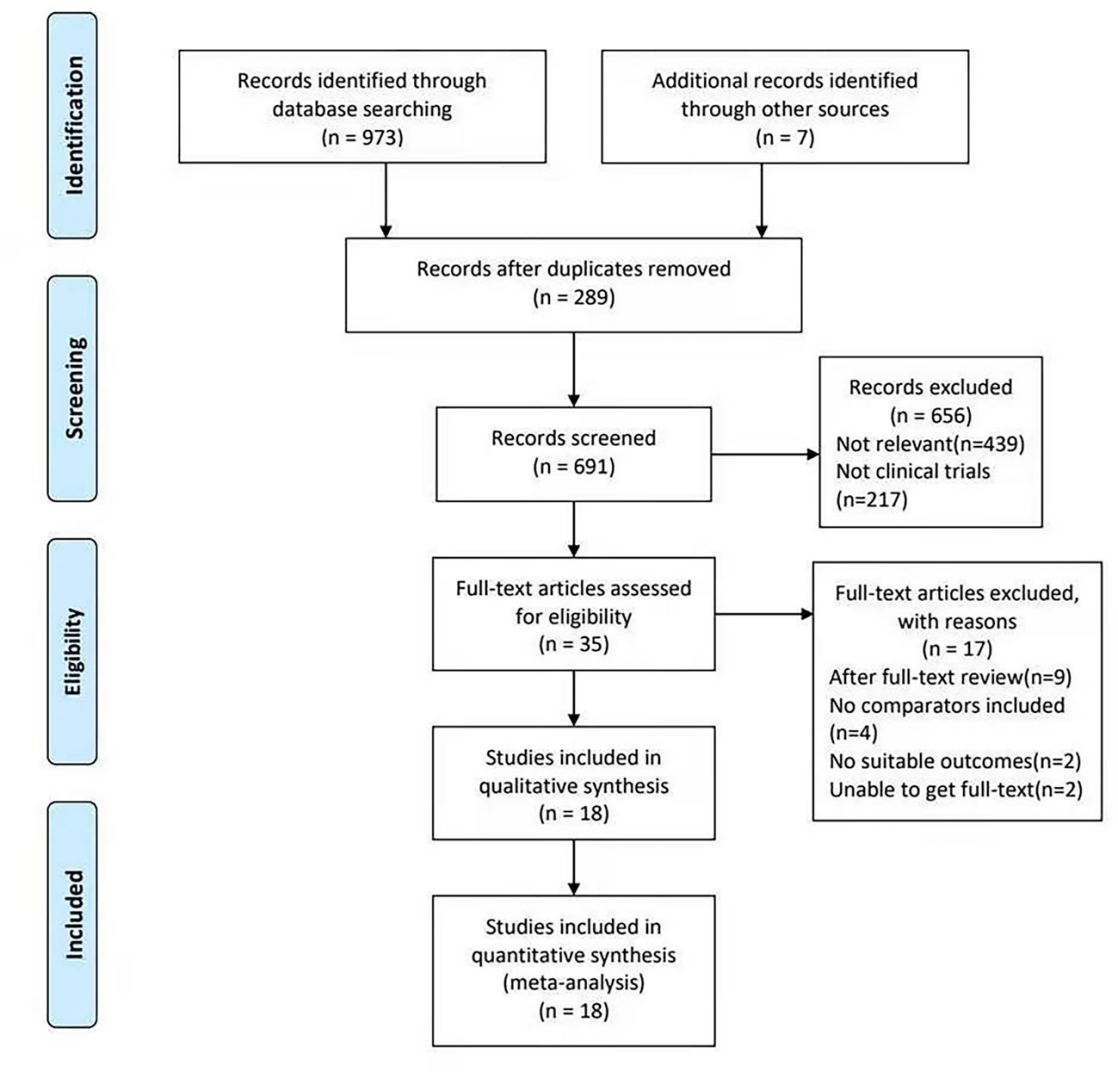
PRISMA Flow Diagram on the literature selection process in this meta-analysis. PRISMA Flow Diagram showing how studies were searched and screened. The flow diagram template was adapted from the 2009 PRISMA statement (28).

The procedure of literature selection is based on the PRISMA statement (28) described in **Figure 1**. In total, 973 records were identified through different databases, of which 7 records were identified after reviewing the reference lists of the retrieved articles. After duplicate studies (n=289) were ruled out, 691 remaining studies have glanced over. After screening the title and abstract, 656 studies were excluded. After reading the full text of the remaining 35 articles carefully, 17 studies were excluded (9 articles were excluded after the full text was reviewed, 4 articles were excluded without comparators included, 2 articles without suitable outcomes, and 2 articles were ruled out for being unable to get full text). Thus, 18 reports (5–22) published between 1982 to 2020 were included in this meta-analysis.

Our study included 422,384 patients. **Table 1** summarizes the basic characteristics of the research included in this systematic review. Of the included studies, 6 were from the USA, 2 were conducted in Greece, and 1 each was from Germany, China, Sweden, Brazil, Czech, Turkey, Italy, Poland, Denmark, and the UK, respectively. The countries were further divided into 2 groups to investigate the region characteristic of BTD on the risk of BC. Greece, Germany, Sweden, Czech, Italy, Poland, Denmark, and the UK belong to the European group. While the USA, China, Brazil, and Turkey belong to the non-European group. The studies varied in sample size from 9 to 139,124. According to the NOS evaluation system (25), all included articles were evaluated with high-quality (**Additional File 1**; **Supplement Figure 2** **).**

**Table 1:** Characteristics of the studies included in the meta-analysis.

First author, year	Region	Study design	Cases	Control	Median/Mean age	Summary of findings
					(years)	
Bach et al. (2020) (5)	Germany	A retrospective case-control study	7480	7408	58.4	Increased risk of BC in thyroiditis patients
Weng et al. (2020) (20)	USA	A prospective cohort study	22382	111740	65	Decreased risk of BC in hypothy-roidism patients
Weng et al. (2018) (6)	Taiwan, China	A retrospective popula-tion-based case-control study	51733	51733	53.3	roidism and hyperthyroidism patients
Freitas et al (2016) (18)	Brazil	A retrospective case-control study	112	125	54	No relationship between the inci-dence of BC and AITD
Søgaard et al (2016) (13)	Denmark	A prospective popula-tion-based cohort study	3092	139124	70.5	Increased risk of BC in hypothy-roidism and hyperthyroidism patients
Tosovic et al (2014) (14)	Sweden	A population-based pro-spective cohort study	149	2036	50.4	T3 positively associated with a high risk of BC
Szychta et al. (2013) (7)	Poland	A retrospective case-control study	9	1630	36.5	TRAb is a positive determinat of BC
Jiskra et al. (2007) (8)	Czech	A Cross-sectional study design	84	49	67	Increased prevalence of AITD as-sociated with BC
Cristofanilli et al. (2005) (16)	USA	A retrospective case-control study	1136	1088	51	Hypothyroidism is associated with a reduced risk of BC
Turken et al. (2003) (9)	Turkey	A Cross-sectional study	150	100	63	Increased prevalence of AITD as-sociated with BC
Simon et al. (2002) (19)	USA	A population-based case-control study	4575	4682	49.5	No relationship between BTD and BC
Gogas et al. (2001) (10)	Greece	A Cross-sectional study	310	190	59	Increased prevalence of AITD as-sociated with BC
Talamini et al. (1997) (20)	Italy	A case-control study	2569	2588	55	No relationship between BTD and BC
Giani et al. (1996) (11)	USA	A Cross-sectional study	102	100	54.3	Increased prevalence of AITD as-sociated with BC
Smyth et al. (1996) (15)	USA	A Cross-sectional study	200	200	57	Increased prevalence of goiter in patients with BC, no relationship between hypothyroidism or hyper-thyroidism
Adamopoulos et al. (1986) (12)	Greece	A prospectively cross-sectional study	97	60	46.7	Increased prevalence of goiter in BC patients
Brinton et al. (1984) (21)	USA	A case-control study	1552	1357	45	No relationship between BTD and BC

BTD, benign thyroid diseases; BC, breast cancer; TPO, thyroid peroxidase; AITD, autoimmune thyroiditis; GD, Graves’ disease.

### BTD increases the risk of BC

**Figure 2:**
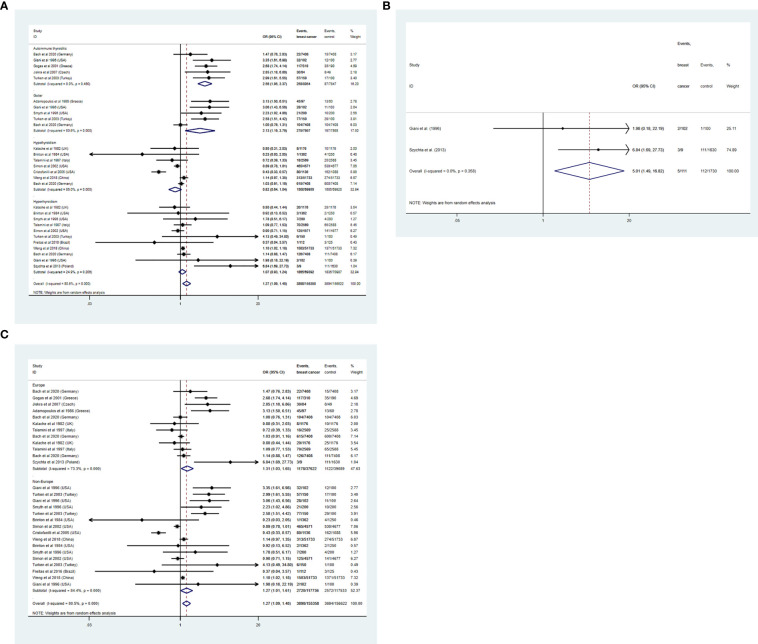
**(A)** A forest plot for assessing the association between BTD and breast cancer. **(B)** A forest plot for assessing the association between GD and breast cancer. **(C)** subgroup analysis of different regions on the BTD and BC risk.

As shown in **Figure 2A**, the present meta-analysis includes 15 studies in total. Our pooled analysis of these reports showed a higher risk of developing BC for people with BTD (OR: 1.27, 95%CI: 1.09–1.48, I^2 =^ 80.5%, n=15). After stratifying by different kinds of BTD, we found that AITD (5, 8–11) (OR: 2.56, 95%CI: 1.95–3.37, I^2 =^ 0.0%, n=5), and goiter (5, 9, 11, 12, 15) (OR: 2.13, 95%CI: 1.19-3.79, I^2 =^ 80.6%, n=5) were positively related to the risk of BC. However, both hypothyroidism (5, 6, 16, 19–22) (OR: 0.82, 95%CI: 0.64-1.04, I^2 =^ 85.0%, n=7) and hyperthyroidism (5–7, 9, 11, 15, 18–22) (OR: 1.07, 95%CI: 0.93-1.24, I^2 =^ 24.9%, n=11) had no correlations with BC risk. GD is the most common cause when considering various causes of hyperthyroidism (29, 30). Hyperthyroid patients with positive TRAb were therefore singled out as the GD group to further investigated the underlying relationship between them. Our result showed a positive correlation between GD (7, 11) (OR: 5.01, 95%CI: 1.49-16.82, I^2 =^ 0.0%, n=2) and BC risk **(** **Figure 2B** **)**. Thus, GD increased the risk of BC was newly proposed in the present study.

**Figure 3:**
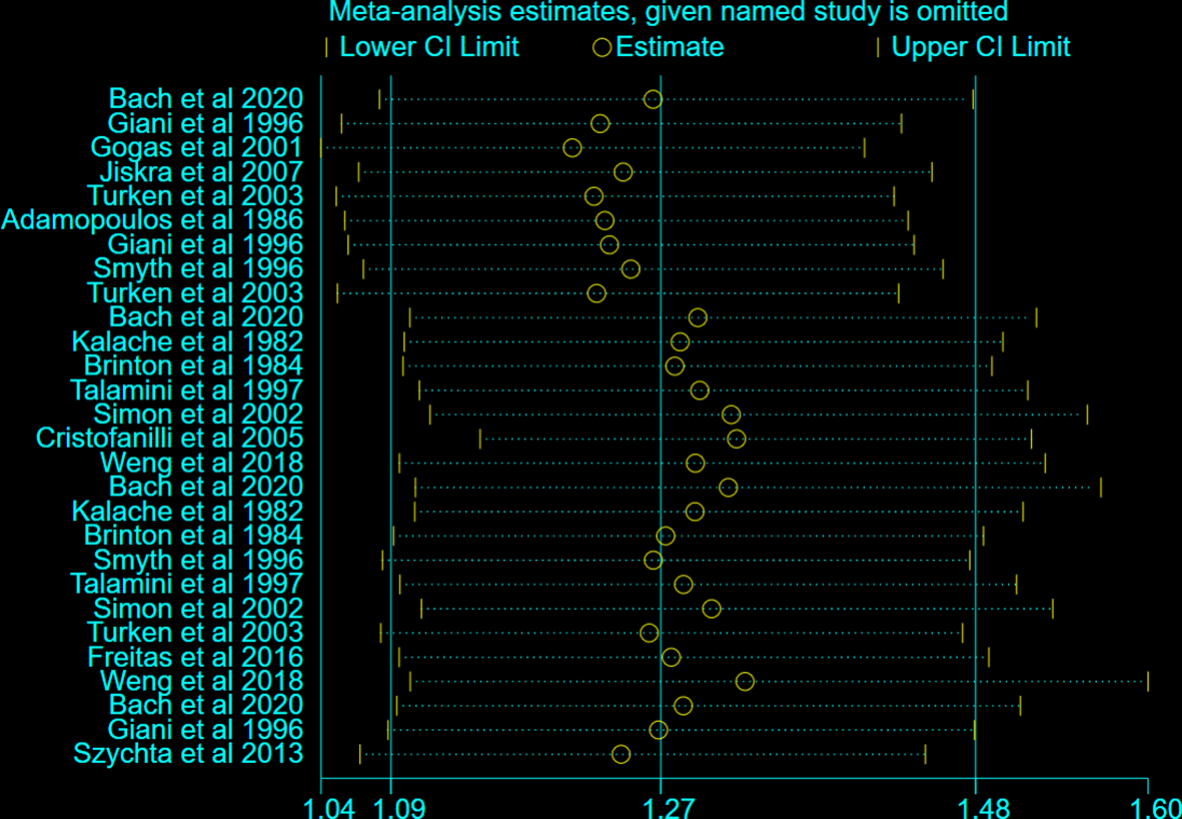
Sensitivity analysis on the BTD and BC risk.

To further explore the heterogeneity between the included studies, subgroup analysis was applied to different regions (Europe and Non-Europe) (**Figure 2C**). BTD had a positive correlation with the risk of BC both in Europe (OR: 1.31, 95%CI: 1.03-1.65, I^2 =^ 73.3%) and Non-Europe (OR: 1.27, 95%CI: 1.01-1.61, I^2 =^ 84.4%) subgroup. However, after stratification by different regions, the considerable heterogeneity did not decrease significantly. In addition, sensitivity analysis was conducted to further explore the sources of heterogeneity. After ignoring individual studies, the considerable heterogeneity did not decrease significantly (**Figure 3**).

### BTD and aggressiveness of BC

**Figure 4:**
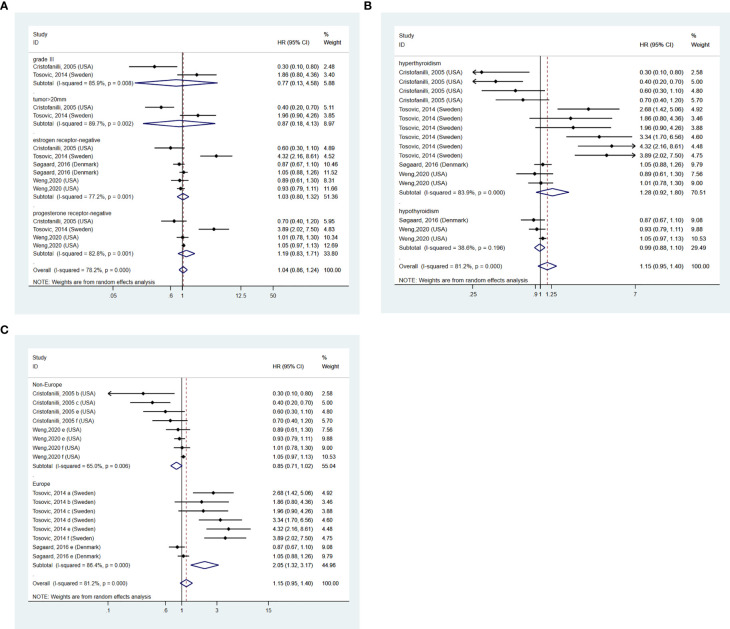
**(A)** BTD and aggressiveness of breast cancer. **(B)** subgroup analysis of different kinds of BTD on and aggressiveness of BC. **(C)** subgroup analysis of different regions on the aggressiveness of BC. a=grade II, b=grade III, c=tumor>20mm, d=lymph gland metastases, e=estrogen receptor negative, f=progesterone receptor negative.

In addition, we further investigated whether the existence of thyroid dysfunction exerted an influence on the aggressiveness of BC (31). A total of 4 pieces of research (13, 14, 16, 17) were included eventually. Subgroup analysis was conducted on different aggressiveness markers of BC (14), grade II, and lymph gland metastases subgroups were excluded for only one article available. Our pooled results demonstrated no relationship between BTD and grade III subgroup (HR: 0.77, 95%CI: 0.13-4.58, I^2 =^ 85.9%, p=0.008, n=2), tumor>20mm subgroup (HR: 0.87, 95%CI: 0.18-4.13, I^2 =^ 89.7%, p=0.002, n=2), estrogen receptor-negative subgroup (HR: 1.03, 95%CI: 0.80-1.32, I^2 =^ 77.2%, p=0.001, n=4) and progesterone receptor-negative subgroup (HR: 1.19, 95%CI: 0.83-1.71, I^2 =^ 82.8%, p=0.001, n=3). The result is shown in **Figure 4A**. 2 studies (13, 17) cohered with our pooled result that there is no significant relation between BTD and the aggressiveness of BC. To future investigated whether there was a difference between different kinds of BTD and aggressiveness of BC, a subgroup analysis was conducted on different kinds of BTD. After retrieving related literature comprehensively, the existing articles mainly focused on hyperthyroidism and hypothyroidism. The present synthesis analysis didn’t find a relationship in hyperthyroidism (HR: 1.28, 95%CI: 0.92-1.80, I^2 =^ 83.9%, p=0.000, n=4) and hypothyroidism (HR: 0.99, 95%CI: 0.88-1.10, I^2 =^ 38.6%, p=0.196, n=2) subgroup (**Figure 4B**). Subgroup analysis based on the study design found a positive relationship between BTD and aggressiveness of BC in the Europe subgroup (HR: 2.05, 95%CI: 1.32-3.17, I^2 =^ 86.4%, p=0.000, n=2) (**Figure 4C**). However, according to subgroup analysis, no decrease in heterogeneity was observed.

### Publication bias

**Figure 5:**
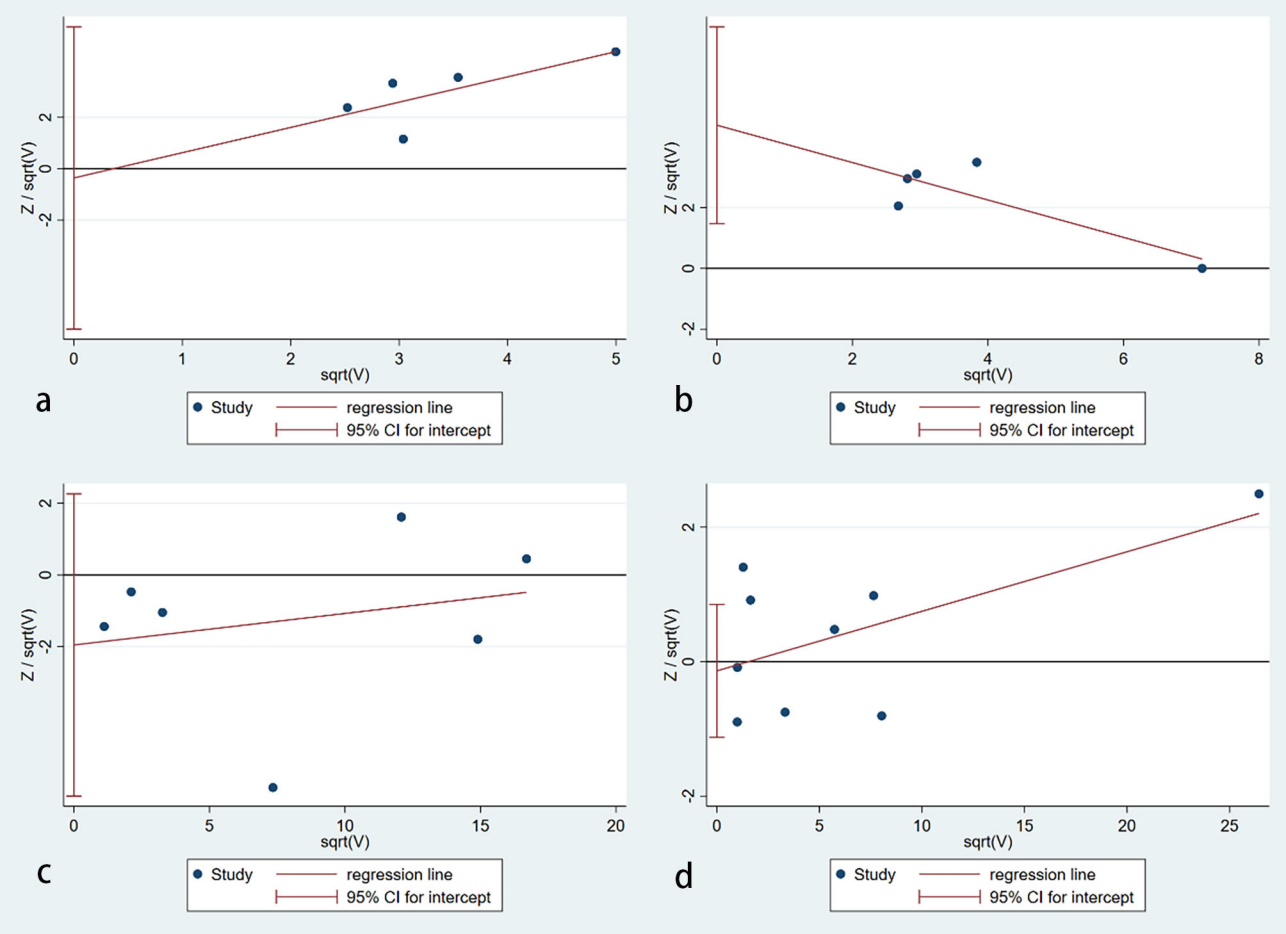
Publication bias assessment. **(A)** an autoimmune thyroiditis; **(B)** goiter; **(C)** hypothyroidism; **(D)** hyperthyroidism (no publication bias was found in autoimmune thyroiditis (p=0.857), hypothyroidism (p=0.287) and hyperthyroidism (p=0.754) subgroups. However, publication bias existed in the goiter subgroup with a p-value of 0.019).

The publication bias detection of the literature included was analyzed using the Harbord test (32) (**Figure 5**). **Figure 5A** shows that the publication bias for the relationship between autoimmune thyroiditis and BC risk was inexistent (p=0.857). Similarly, no publication bias was observed in the hypothyroidism (p=0.287) and hyperthyroidism (p=0.754) subgroups. However, publication bias existed in the goiter subgroup with a p-value of 0.019. To verify the reliability of the result of the meta-analysis in the goiter subgroup, sensitivity analysis (33) was used. After removing the study reported by Bach et al (5), publication bias is inexistent with a p-value of 0.949. Other sensitivity analysis results were consistent with the preliminary analysis.

## Discussion

### Relationship between BTD and the risk of BC

The current systematic review summarized and combined the available data on the relationship between BTD and the risk of BC in females. Our results from a meta-analysis of the eligible studies showed that ATID and goiter increased the risk of BC, which was consistent with previous studies. Additionally, we found that GD was related to an increased risk of BC in the current meta-analysis. Furthermore, subgroup analysis based on the study region (Europe and Non-Europe) suggested a positive relationship between BTD and aggressiveness of BC in the Europe subgroup. However, as a high degree of heterogeneity was observed among studies, the current results may be biased.

### Underling mechanisms between thyroid autoantibody and the development of BC

A significant feature of AITD is the existence of autoantibodies, including TPO-Ab, TgAb, and microsomal. However, the diagnosis of AITD not only relies on the presence of autoantibodies but also needs evidence of thyroid dysfunction or histological confirmation (34). There was no denying that TPO-Ab plays a significant role in the development of AITD. Based on the existing evidence, TPO-Ab may be a protective factor for BC and a higher TPO-Ab level was associated with a lower risk of BC (35, 36). A higher TPO-Ab level at baseline corresponds with autoimmune thyroiditis. What happens next is the development of hypothyroid with a low level of thyroid hormone. It will eventually play a protective role in developing BC. Although many studies concluded a relationship between hypothyroidism and the following lower risk of BC (37, 38), our synthesized analysis didn’t reach statistically significant. This result was similar to the creation of Wang et al (39). Prospective studies have shown that women with high TPO-Ab levels have a lower risk of being diagnosed with invasive BC (35). It shouldn’t be ignored that a large number of cross-sectional studies (10) and existing meta-analysis (40) has proved that there is a positive correlation between the level of TPO-Ab and BC, one possible explanation is that we never know whether BC itself stimulated the elevating of TPO-Ab and it was the main drawback of cross-sectional studies. Based on existing evidence, the relationship between AITD and BC remained controversial (41). Cohort studies based on large numbers of the population were indispensable to draw a firm conclusion.

### Underlying mechanisms between hyperthyroidism and the development of BC

In the present study, the relationship between hyperthyroidism and the risk of BC has not been concluded. After comparing with previous studies which focus on hyperthyroidism and the risk of BC roundly, we found that the studies which were added in the present study were based on a large population-based dataset and it can minimize the selection bias. However, firm conclusions about them still can’t be drawn based on existing research. Although our pooled analysis didn’t reach a statistical correlation, it was undeniable that there is a link between hyperthyroidism and the risk of BC. Many studies concluded that a high pre-diagnostic fT4 level was positively associated with a high risk of BC (35, 36, 42). Sterle et al. found that thyroid hormones regulated BC cell proliferation in two ways, directly or through the cellular and cytokine content of the tumor microenvironment and the systemic immunity in the mouse model (43). Besides, 2 prospective cohort studies (13, 44) confirmed that hyperthyroidism increased the risk of BC, but they were excluded from this study for only IRR or HIR attainable.

The potential mechanisms between hyperthyroidism and BC have been studied for a long period. Several hypotheses have been suggested. It was widely accepted that sodium iodide symporter (NIS) existed both in thyroid and BC tissue and an increased expression of the NIS in BC tissue was already demonstrated (45, 46). For the absorption and oxidation of iodine, NIS participates and plays a role in the progress of BC. A study by Dong et al. (47) hypothesized that incorrect positioning of NIS protein may lead to abnormal NIS expression. It will soon result in iodine deficiency, which can stimulate the secretion of gonadotropin. The over-production of gonadotropin led to high estrogen status, and such changes in endocrine status may increase the risk of BC and thyroid diseases. The interaction between the thyroid and mammary gland mainly involves the triiodothyronine (T3) and thyroxine (T4) pathways, and then in an estrogen-like manner activate the thyroid hormone receptors and induced differentiation and lobular growth of the mammary gland (48, 49). Besides, overweight or obese (BMI>25kg/m^2^) women with high fT4 were more likely to develop BC than normal-weight women, for the estrogen level of obese women is higher than that of normal-weight women (36). Thyroid hormones can enhance the effects of estrogens on BC proliferation and estrogens may act on the same receptors as thyroid hormones (50). Interestingly, a study by Jonklaas et al (51) found malignancy was associated with the occurrence of hyperthyroidism. As an endocrine gland, the thyroid gland may become the target of metastases of several non-endocrine cancers because of its abundant blood supply. It led to damage or destruction of thyroid tissue, started with hyperthyroidism, and then turned to hypothyroidism in the end.

Compared with the existing meta-analysis, we added the results between GD and the risk of BC. In the present meta-analysis, GD is considered positively associated with the risk of BC. GD is the most common cause of hyperthyroidism. It is already known that thyroid-stimulating antibodies (TSAb) are the primary cause of Graves’ hyperthyroidism. TSH stimulates the growth, differentiation, and function of the thyroid cells *via* TSHR and is a target for TSAb in the development of GD. At the same time, the expression of TSHR was found common in BC, especially with a higher prevalence in low-grade breast cancer (52). A growing number of experimental researches confirmed that TSH-R is expressed in several non-thyroid cells, such as murine and human normal and BC tissues (41). Davies et al (53) found TSH receptors are abundant in the fatty tissue of the mammary gland and the mutual effect between the thyroid gland and breast tissue was explained to some extent. Several relevant studies had already demonstrated that GD increased the risk of BC and this conclusion was consistent with our meta-analysis. However, the sample size of included research is small, more studies based on a large sample were needed to draw a more convincing result. In brief, the result of the present study was the most convincing for the most comprehensive literature included.

### BTD and aggressiveness of BC

This meta-analysis further studies the relationship between BTD and the aggressiveness of BC. Larger tumors, negative ER and PGR status, and the appearance of lymph node metastases all indicated aggressiveness. Although some studies indicated that a history of hyperthyroidism was associated with an increased risk of invasive BC and hypothyroidism was related to a lower risk of BC, synthesis analysis did not reach statistical significance. The study of Tosovic et al (14) found hyperthyroidism significantly increased the risk of developing more aggressive BC, while Cristofanilli et al (16) showed less aggressive BC among hypothyroid patients.

Due to the limited quantity and high heterogeneity of studies incorporated in this meta-analysis, we can’t draw a precise conclusion. Subgroup analysis was conducted by different aggressiveness markers of BC, different kinds of BTD, and regions. However, we didn’t find a valuable factor that could be used as a parameter to decrease the heterogeneity in any subgroup. The existing research did not provide enough data on the parameters such as age, sex, and menopausal status at the diagnosis of BC, they are not compatible with the present study. Among the results we obtained in the present study, we found that BTD increases the risk of BC in the Europe subgroup. The possible reasons for the disparity may be different gene-gene and gene-environmental backgrounds which come from different ethnicities. Because of the high prevalence and mortality of BC in women, it is of great value to fully understand the risk factors and aggressiveness factors and to do primary prevention. The existing evidence gave us a direction to further conduct more prospective studies to explore the influence of BTD on the aggressiveness of BC and it is necessary to conduct basic research to clarify how BTD exerted an impact on the aggressiveness of BC.

### Limitation

Several limitations should be acknowledged. Firstly, the majority of the studies included in our meta-analysis are cross-sectional studies. In the cross-sectional study, it is difficult to determine the causal relationship between BTD and BC. Secondly, the quantity of articles included is limited, especially in the GD subgroup. Thus, more prospective studies were needed to further illustrate the exact relationship between BTD and BC risk. Thirdly, due to the limited number of researches, our meta-analysis only analyzed ER positive, PR positive, grade III, and tumor>20mm subgroup in the section of BTD and the aggressiveness of BC. More studies were needed for a more comprehensive conclusion including Triple-negative, HER2 positive subgroup. In addition, there is publication bias that existed in the goiter subgroup of our meta-analysis. One possible explanation for this phenomenon is that the subgroup of our article contains a small number of studies.

## Conclusion

This systematic review summarized available studies on the association between BTD and the risk of BC in females. Our results found that AITD, goiter, and GD were related to the increased risk of BC. Additionally, BTD is connected with an increased risk of aggressiveness of BC in the European population. Therefore, we should pay particular attention to these patients during treatment and follow-up. These positive observations are weak as the available studies are limited, however, deserve further investigation.

## Data availability statement

The original contributions presented in the study are included in the article/**Supplementary Material**. Further inquiries can be directed to the corresponding authors.

## Author contributions

MH, XG, and GW designed the study. MH and YW wrote the paper. MH and XZ selected the paper. XZ, YJ, and HC did the data extraction and analysis. All authors read and approved the final manuscript.

## Funding

This work was supported by grants from the National Natural Science Foundation of China (81972372 to XG, 82272993 to XG, 81970687 to GW), the grant from Department Science and Technology Department of Jilin Province (20190901006JC to GW and YDZJ202202CXJD042 to GW), and the grant from Jilin Medical and Health Talent Project (JLSCZD2019-016 to GW).

## Conflict of interest

The authors declare that the research was conducted in the absence of any commercial or financial relationships that could be construed as a potential conflict of interest.

## Publisher’s note

All claims expressed in this article are solely those of the authors and do not necessarily represent those of their affiliated organizations, or those of the publisher, the editors and the reviewers. Any product that may be evaluated in this article, or claim that may be made by its manufacturer, is not guaranteed or endorsed by the publisher.

## Abbreviations

BTD, benign thyroid disease; BC, breast cancer; AITD, autoimmune thyroiditis; GD, Graves’ Disease; NIS, Sodium iodide symporter; TSAb, Thyroid-stimulating antibodies; OR, Odds Ratio; CI, Confidence Intervals; HR, Hazard Ratio; NOS, Newcastle-Ottawa Scale.

## References

[1] Momenimovahed Z Salehiniya H . Epidemiological characteristics of and risk factors for breast cancer in the world. Breast Cancer (2019) 11:151–64. (Dove Med Press). doi: 10.2147/BCTT.S176070 PMC6462164 31040712

[2] International agency for research on cancer. Available at: https://www.iarc.fr/faq/latest-global-cancer-data-2020-qa/.

[3] Majeed W Aslam B Javed I Khaliq T Muhammad F Ali A . Breast cancer: major risk factors and recent developments in treatment. Asian Pac J Cancer Prev (2014) 15(8):3353–8. doi: 10.7314/APJCP.2014.15.8.3353 24870721

[4] Schottenfeld D . The relationship of breast cancer to thyroid disease. J Chronic. Dis (1968) 21(5):303–13. doi: 10.1016/0021-9681(68)90039-8 4175318

[5] Bach L Kostev K Schiffmann L Kalder M . Association between thyroid gland diseases and breast cancer: a case-control study. Breast Cancer Res Treat (2020) 182(1):207–13. doi: 10.1007/s10549-020-05675-6 32424720

[6] Weng CH Chen YH Lin CH Luo X Lin TH . Thyroid disorders and breast cancer risk in Asian population: a nationwide population-based case-control study in Taiwan. BMJ Open (2018) 8(3):e020194. doi: 10.1136/bmjopen-2017-020194 PMC5884336 29602850

[7] Szychta P Szychta W Gesing A Lewiński A Karbownik-Lewińska M . TSH receptor antibodies have predictive value for breast cancer–retrospective analysis. Thyroid Res (2013) 6(1):8. doi: 10.1186/1756-6614-6-8 23680448 PMC3662571

[8] Jiskra J Barkmanova J Limanova Z Lánská V Smutek D Potlukova E . Thyroid autoimmunity occurs more frequently in women with breast cancer compared to women with colorectal cancer and controls but it has no impact on relapse-free and overall survival. Oncol Rep (2007) 18(6):1603–11. 17982651

[9] Turken O Narin Y Demirbas S Onde ME Sayan O KandemIr EG . Breast cancer in association with thyroid disorders. Breast Cancer Res (2003) 5(5):1–4. doi: 10.1186/bcr609 12927040 PMC314421

[10] Gogas J Kouskos E Tseleni-Balafouta S Markopoulos C Revenas K Gogas G . Autoimmune thyroid disease in women with breast carcinoma. Eur J Surg Oncol (2001) 27(7):626–30. doi: 10.1053/ejso.2001.1204 11669589

[11] Giani C Fierabracci P Bonacci R Gigliotti A Campani D De Negri F . Relationship between breast cancer and thyroid disease: relevance of autoimmune thyroid disorders in breast malignancy. J Clin Endocrinol Metab (1996) 81(3):990–4. doi: 10.1210/jcem.81.3.8772562 8772562

[12] Adamopoulos DA Vassilaros S Kapolla N Papadiamantis J Georgiakodis F Michalakis A . Thyroid disease in patients with benign and malignant mastopathy. Cancer (1986) 57(1):125–8. doi: 10.1002/1097-0142(19860101)57:1<125::AID-CNCR2820570125>3.0.CO;2-4 3940612

[13] Søgaard M Farkas DK Ehrenstein V Jørgensen JO Dekkers OM Sørensen HT . Hypothyroidism and hyperthyroidism and breast cancer risk: a nationwide cohort study. Eur J Endocrinol (2016) 174(4):409–14. doi: 10.1530/EJE-15-0989 26863886

[14] Tosovic A Bondeson AG Bondeson L Ericsson UB Manjer J . T3 levels in relation to prognostic factors in breast cancer: a population-based prospective cohort study. BMC Cancer (2014) 14(1):1–10. doi: 10.1186/1471-2407-14-536 25060772 PMC4131035

[15] Smyth P Smith D McDermott E Murray MJ Geraghty JG O'Higgins NJ . A direct relationship between thyroid enlargement and breast cancer. J Clin Endocrinol Metab (1996) 81(3):937–41. doi: 10.1210/jcem.81.3.8772554 8772554

[16] Cristofanilli M Yamamura Y Kau SW Bevers T Strom S Patangan M . Thyroid hormone and breast carcinoma: primary hypothyroidism is associated with a reduced incidence of primary breast carcinoma. Cancer (2005) 103(6):1122–8. doi: 10.1002/cncr.20881 15712375

[17] Weng C-H Okawa ER Roberts MB Park SK Umbricht CB Manson JE . Breast cancer risk in postmenopausal women with medical history of thyroid disorder in the women's health initiative. Thyroid (2020) 30(4):519–30. doi: 10.1089/thy.2019.0426 PMC7187984 31918623

[18] Freitas PA Vissoci GM Pinto RM Lajolo PP Jorge PT . Study of the prevalence of autoimmune thyroid disease in women with breast cancer. Endocr Pract (2016) 22(1):16–21. doi: 10.4158/EP14445.OR 26401580

[19] Simon MS Tang M-TC Bernstein L Norman SA Weiss L Burkman RT . Do thyroid disorders increase the risk of breast cancer. Cancer Epidemiol Biomarkers Prev (2002) 11(12):1574–8. 12496046

[20] Talamini R Franceschi S Favero A Negri E Parazzini F La Vecchia C . Selected medical conditions and risk of breast cancer. Br J Cancer (1997) 75(11):1699–703. doi: 10.1038/bjc.1997.289 PMC2223528 9184190

[21] Brinton LA Hoffman DA Hoover R Fraumeni JF Jr . Relationship of thyroid disease and use of thyroid supplements to breast cancer risk. J Chronic Dis (1984) 37(12):877–83. doi: 10.1016/0021-9681(84)90062-6 6526927

[22] Kalache A Vessey M Mcpherson KJ . Thyroid disease and breast cancer: findings in a large case-control study. Br J Surg (1982) 69(7):434–5. doi: 10.1002/bjs.1800690731 7104624

[23] Hardefeldt PJ Eslick GD Edirimanne S . Benign thyroid disease is associated with breast cancer: a meta-analysis. Breast Cancer Res Treat (2012) 133(3):1169–77. doi: 10.1007/s10549-012-2019-3 22434524

[24] Schünemann HJ Lerda D Quinn C Follmann M Alonso-Coello P Rossi PG . Breast cancer screening and diagnosis: a synopsis of the European breast guidelines. Ann Intern Med (2020) 172(1):46–56. doi: 10.7326/M19-2125 31766052

[25] Stang A . Critical evaluation of the Newcastle-Ottawa scale for the assessment of the quality of nonrandomized studies in meta-analyses. Eur J Epidemiol (2010) 25(9):603–5. doi: 10.1007/s10654-010-9491-z 20652370

[26] Higgins JP Thompson SG Deeks JJ Altman DG . Measuring inconsistency in meta-analyses. BMJ (2003) 327(7414):557–60. doi: 10.1136/bmj.327.7414.557 PMC192859 12958120

[27] Huedo-Medina TB Sánchez-Meca J Marín-Martínez F Botella J . Assessing heterogeneity in meta-analysis: Q statistic or I² index. Psychol Methods (2006) 11(2):193. doi: 10.1037/1082-989X.11.2.193 16784338

[28] Moher D Shamseer L Clarke M Ghersi D Liberati A Petticrew M . Preferred reporting items for systematic review and meta-analysis protocols (PRISMA-p) 2015 statement. Syst Rev (2015) 4(1):1–9. doi: 10.1186/2046-4053-4-1 25554246 PMC4320440

[29] Davies TF Andersen S Latif R Nagayama Y Barbesino G Brito M . Graves’ disease. Nat Rev Dis Primers (2020) 6(1):52 doi: 10.1038/s41572-020-0184-y 32616746

[30] Antonelli A Ferrari SM Ragusa F Elia G Paparo SR Ruffilli I . Graves’ disease: Epidemiology, genetic and environmental risk factors and viruses. Best Pract Res Clin Endocrinol Metab (2020) 34(1):101387. doi: 10.1016/j.beem.2020.101387 32107168

[31] Anastasiadi Z Lianos GD Ignatiadou E Harissis HV Mitsis M . Breast cancer in young women: an overview. Updates Surg (2017) 69(3):313–7. doi: 10.1007/s13304-017-0424-1 28260181

[32] Furuya-Kanamori L Xu C Lin L Doan T Chu H Thalib L . P value–driven methods were underpowered to detect publication bias: analysis of cochrane review meta-analyses. J Clin Epidemiol (2020) 118:86–92. doi: 10.1016/j.jclinepi.2019.11.011 31743750

[33] Mathur MB Vanderweele TJ . Sensitivity analysis for publication bias in meta-analyses. J R Stat Soc Ser C Appl Stat (2020) 69(5):1091–119. doi: 10.1111/rssc.12440 PMC7590147 33132447

[34] Ragusa F Fallahi P Elia G Gonnella D Paparo SR Giusti C . Hashimotos’ thyroiditis: Epidemiology, pathogenesis, clinic and therapy. Best Pract Res Clin Endocrinol Metab (2019) 33(6):101367. doi: 10.1016/j.beem.2019.101367 31812326

[35] Brandt J Borgquist S Manjer J . Prospectively measured thyroid hormones and thyroid peroxidase antibodies in relation to risk of different breast cancer subgroups: a malmo diet and cancer study. Cancer Causes Control (2015) 26(8):1093–104. doi: 10.1007/s10552-015-0602-8 26033776

[36] Tosovic A Becker C Bondeson AG Bondeson L Ericsson UB Malm J . Prospectively measured thyroid hormones and thyroid peroxidase antibodies in relation to breast cancer risk. Int J Cancer (2012) 131(9):2126–33. doi: 10.1002/ijc.27470 22323002

[37] Liu Y-C Yeh C-T,Lin K-H . Molecular functions of thyroid hormone signaling in regulation of cancer progression and anti-apoptosis. Int Mol Sci J (2019) 20(20):4986. doi: 10.3390/ijms20204986 PMC7279030 32438653

[38] Hercbergs A Mousa SA Leinung M Lin HY Davis PJ . Thyroid hormone in the clinic and breast cancer. Horm Cancer (2018) 9(3):139–43. doi: 10.1007/s12672-018-0326-9 PMC5945724 29441459

[39] Wang B Lu Z Huang Y Li R Lin T . Does hypothyroidism increase the risk of breast cancer: evidence from a meta-analysis. BMC Cancer (2020) 20(1):733. doi: 10.1186/s12885-020-6564-6 32762667 PMC7409635

[40] Chen S Wu F Hai R You Q Xie L Shu L . Thyroid disease is associated with an increased risk of breast cancer: a systematic review and meta-analysis. Gland Surg (2021) 10(1):336–46. doi: 10.21037/gs-20-878 PMC7882351 33633990

[41] Baldini E Lauro A Tripodi D Pironi D Amabile MI Ferent IC . Thyroid diseases and breast cancer. J Pers Med (2022) 12(2):156. doi: 10.3390/jpm12020156 35207645 PMC8876618

[42] Khan SR Chaker L Ruiter R Aerts JG Hofman A Dehghan A . Thyroid function and cancer risk: the Rotterdam study. J Clin Endocrinol Metab (2016) 101(12):5030–6. doi: 10.1210/jc.2016-2104 27648963

[43] Sterle HA Hildebrandt X Valenzuela Alvarez M Paulazo MA Gutierrez LM Klecha AJ . Thyroid status regulates the tumor microenvironment delineating breast cancer fate. Endocr Relat Cancer (2021) 28(7):403–18. doi: 10.1530/ERC-20-0277 33908371

[44] Yang H Holowko N Grassmann F Eriksson M Hall P Czene K . Hyperthyroidism is associated with breast cancer risk and mammographic and genetic risk predictors. BMC Med (2020) 18(1):1–10. doi: 10.1186/s12916-020-01690-y 32838791 PMC7446157

[45] Tazebay UH Wapnir IL Levy O Dohan O Zuckier LS Zhao QH . The mammary gland iodide transporter is expressed during lactation and in breast cancer. Nat Med (2000) 6(8):871–8. doi: 10.1038/78630 10932223

[46] Kogai T Taki K Brent G J E-R C . Enhancement of sodium/iodide symporter expression in thyroid and breast cancer. Endocr Relat Cancer (2006) 13(3):797–826. doi: 10.1677/erc.1.01143 16954431

[47] Dong L Lu J Zhao B Wang W Zhao Y . Review of the possible association between thyroid and breast carcinoma. World J Surg Oncol (2018) 16(1):1–7. doi: 10.1186/s12957-018-1436-0 29976206 PMC6034293

[48] Conde I Paniagua R Zamora J Blánquez MJ Fraile B Ruiz A . Influence of thyroid hormone receptors on breast cancer cell proliferation. Ann Oncol (2006) 17(1):60–4. doi: 10.1093/annonc/mdj040 16282247

[49] González-Sancho JM Garcıía V Bonilla F Muñoz A . Thyroid hormone receptors/THR genes in human cancer. Cancer Lett (2003) 192(2):121–32. doi: 10.1016/s0304-3835(02)00614-6 12668276

[50] Hall LC Salazar EP Kane SR Liu N . Effects of thyroid hormones on human breast cancer cell proliferation. J Steroid Biochem Mol Biol (2008) 109(1-2):57–66. doi: 10.1016/j.jsbmb.2007.12.008 18328691

[51] Jonklaas J . Infiltration of the thyroid gland by non-thyroid malignancy: A literature review reveals this to be an unusual cause of hyperthyroidism. J Clin Transl Endocrinol (2020) 20:100221. doi: 10.1016/j.jcte.2020.100221 32154117 PMC7052397

[52] Oh HJ Chung J-K Kang JH Kang WJ Noh DY Park IA . The relationship between expression of the sodium/iodide symporter gene and the status of hormonal receptors in human breast cancer tissue. Cancer Res Treat (2005) 37(4):247. doi: 10.4143/crt.2005.37.4.247 19956522 PMC2785920

[53] Davies TF METABOLISM . The thyrotropin receptors spread themselves around. J Clin Endocrinol Metab (1994) 79(5):1232–3. doi: 10.1210/jcem.79.5.7962313 7962313

